# Sexuality and marital satisfaction in patients followed for breast cancer in a regional hospital

**DOI:** 10.1192/j.eurpsy.2023.2002

**Published:** 2023-07-19

**Authors:** F. Zaouali, I. Abbes, A. Ben Slama, I. Belhaj Youssef

**Affiliations:** 1 Outpatient Medical Oncology consultation, Haj Ali Soua regional hospital, Ksar Hellal, Monastir; 2 Family Medicine Department, University of Monastir, Monastir; 3 Outpatient Physical Medicine and Rehabilitation consultation, Haj Ali Soua regional hospital, Ksar Hellal, Monastir, Tunisia

## Abstract

**Introduction:**

Breast cancer is the leading cancer in women in developed and developing countries. Treatment strategies can affect sexuality in the short or long term.

**Objectives:**

The aim of our study was to assess sexuality and martial satisfaction in patients followed for breast cancer.

**Methods:**

Cross-sectional descriptive study including patients followed for breast cancer at the outpatient medical oncology consultation of Hadj Ali Soua regional hospital from January to March 2021. We passed the Female Sexual Function Index (FSFI) and the martial adjustment test (MAT).

**Results:**

Fifteen patients were included with a mean age of 49.87 ± 8.48 years and a mean age at diagnosis of 46.73 ± 7.55 years. At the TNM classification, 66.6% of the patients had a T1 or T2 at the time of diagnosis and 80% had an N0. All patients received a surgical intervention, which was conservative in 53.3% of cases. No patient underwent breast reconstruction. Chemotherapy and hormone therapy were prescribed in 86.7% of patients. Radiotherapy and targeted therapy were prescribed in 12 and 2 case, respectively. Amenorrhea, hot flushes and vaginal dryness were noted in 98.7%, 26.7% and 7.6% of patients, respectively. Sexual disorders were found in 53.3% of cases, which settled in a chronic mode in 75% of cases and progressed in a continuous mode in half of cases. The assessment of physical and erotic life was revealed to be neat in 73.3% of the patients. The mean score of the FSFI questionnaire was 17.25 [2.6-31.9]. Eleven patients (73.3%) had sexual dysfunction. A low marital satisfaction was found in 34% of cases.

**Conclusions:**

The medical consultation to identify sexuality disorders in correlation with the martial dissatisfaction in women followed for new breast cancer is crucial allowing a better management of this pathology.

**Disclosure of Interest:**

None Declared

